# Administration of routine preventative vaccinations in children with polyarticular juvenile idiopathic arthritis receiving adalimumab

**DOI:** 10.1186/1546-0096-12-S1-P20

**Published:** 2014-09-17

**Authors:** Kirsten Minden, Mahinda Karunaratne, Hartmut Kupper, Jasmina Kalabic

**Affiliations:** 1 Kinderklinik der Charite, Otto-Heubner Centrum, Berlin, Germany; 2 AbbVie Inc, North Chicago, United States; 3 AbbVie Deutschland GmbH & Co. KG, Ludwigshafen, Germany

## Introduction

Adalimumab (ADA) has been shown to be safe and effective in polyarticular juvenile idiopathic arthritis (pJIA), and is approved for use in moderate to severe pJIA patients (pts) ≥4 years (yrs) in the US, Australia, and Japan, and in the EU for pts ≥2 yrs.

## Objectives

This *post hoc* report describes the observed use of vaccines in pJIA pts receiving ADA in 3 clinical trials and one registry.

## Methods

Pts with active pJIA were enrolled in one of the following trials: M10-444 (ages 2 to <4 or ≥4 weighing <15 kg in US, EU), M10-240 (ages 4-17 in Japan), DE038 (ages 4-17 in US, EU) or the STRIVE (P10-262) registry (ages 4-17 in US, EU, and Australia). Pts received ADA±methotrexate. Vaccinations were administered based on the judgment of the study investigator or the treating physician. Descriptive statistics were used to summarize all vaccinations. Adverse events (AEs) related to active influenza virus infection events occurring within 270 days after influenza vaccination were collected by a predefined MedDRA query 15.1 [Lack of efficacy/effect Influenza (Vaccination Product Specific)].

## Results

The influenza vaccine was the most frequently administered: 55, 63, 10 and 22 influenza vaccines were administered in DE038, M10-240, M10-444 and P10-262, respectively. In addition, pneumococcal, human papilloma virus, diphtheria, tetanus and/or pertussis, hepatitis A and B, and polio vaccines were administered. 2 pts each received >5 vaccinations in DE038 and M10-240, while 3 pts each in M10-444 and P10-262 received >1 but <5 vaccinations. The influenza vaccine was administered to 32/171 (19%), 20/25 (80%), 6/32 (19%) and 21/533 (4%) of pts during the course of the study in DE038, M10-240, M10-444 and P10-262 respectively, and the mean (SD) time to 1st influenza vaccination while pts were on ADA was high: 675 (618), 189 (80), 93 (90) and 443 (396) days. The rates of influenza-related AEs reported for pts who received influenza vaccinations and those who did not were: 13% vs 9% for DE038, 15% vs 20% for M10-240, 0% vs 12% for M10-444, and 5% vs 0.4% for P10-262. Table 1.

**Table 1:** 

All Vaccinations	DE038	M10-240	M10-444	P10-262
Total patients vaccinated, n/N	38/171	20/25	6/32	22/533

Mean age, yrs (SD)	11.8 (3.6)	13.6 (3.3)	3.0 (0.7)	12.7 (4.0)

Total vaccinations, n	77	64	10	25

Patients with >1 type of vaccination, n	11	1	0	2

Different types of vaccinations, n	9	2	1	4

Mean time to 1^st^ vaccination*, days	688	189	93	448

Mean age at 1^st^ vaccination*, yrs	12.9	14.0	3.6	12.0

^*while on ADA^

## Conclusion

These data support the idea that pJIA pts treated with ADA can be immunized with routine, inactive, preventative vaccines. Not all of the eligible pts were vaccinated on time according to the Centers of Disease Control (CDC) recommendations, and many pts were not vaccinated at all, suggesting that physicians may be reluctant to use vaccines in children receiving antirheumatic therapies. Further investigation of vaccination practices for pts with JIA is warranted.

## Trial registration identifying numbers

NCT00774537, NCT00690573, NCT00048542 and NCT00783510

## Disclosure of interest

K. Minden Grant / Research Support from: AbbVie, Pfizer, Consultant for: Pfizer, AbbVie, Roche/Chugai, Novartis, Medac, Pharm-Allergan, Speaker Bureau of: Pfizer, AbbVie, Roche/Chugai, Novartis, Medac, Pharm-Allergan, M. Karunaratne Shareholder of: AbbVie, Employee of: AbbVie, H. Kupper Shareholder of: AbbVie, Employee of: AbbVie, J. Kalabic Shareholder of: AbbVie, Employee of: AbbVie

